# Supplementary material for: Coagulation profile of COVID-19 patients admitted to the ICU: An exploratory study
Source: PLoS One. 2020 Dec 15;15(12):e0243604. doi: 10.1371/journal.pone.0243604 (PMC7737963; doi:10.1371/journal.pone.0243604)
Supplement: S1 Table — (DOCX) [file pone.0243604.s001.docx]

**S1 Table.** Fibrinolysis and endogenous inhibitors of coagulation.

| **Parameters** | **Reference range** | **Day 0** | **Day 1** | **Day 3** | **Day 7** | **Day 14** | **P value** |
| --- | --- | --- | --- | --- | --- | --- | --- |
| D-dimer (ng/ml) | <500 |  |  |  |  |  |  |
| All patients |  | 1287 (798-2202) | 1787 (762-4048) | 1736 (1319-4192) | 3444 (1715-4695) | 2319 (1055-4404) | 0.221^a^ |
| SOFA$\leq10$ |  | 1455 (690-2693) | 1693 (707-2960) | 1419 (846-4380) | 2515 (1470-4624) | 1472 (701-2672) | 0.279^b^ |
| SOFA >10 |  | 1287 (968-2160) | 1913 (968-5517) | 1783 (1578-4192) | 4273 (3045-6205) | 3794 (2178-5528) | 0.272^c^ |
| Plasminogen (%) | 80-132 |  |  |  |  |  |  |
| All patients |  | 85 (70-101) | 86 (74-96) | 89 (71-107) | 103 (82-123)* | 106 (85-128)* | <0.001^a^ |
| SOFA$\leq10$ |  | 88 (80-108) | 92 (85-110) | 102 (82-113) | 113 (96-138) | 117 (94-129) | 0.010^b^ |
| SOFA >10 |  | 83 (65-90) | 74 (68-88)^#^ | 70 (64-88)^#^ | 94 (73-116) | 88 (84-112) | 0.363^c^ |
| Alpha-2 antiplasmin (%) | 98-122 |  |  |  |  |  |  |
| All patients |  | 129 (112-151) | 127 (112-139) | 128 (116-139) | 126 (115-138) | 119 (113-128) | 0.405^a^ |
| SOFA$\leq10$ |  | 136 (126-152) | 132 (123-147) | 128 (115-139) | 131 (117-138) | 123 (108-132) | 0.166^b^ |
| SOFA >10 |  | 124 (109-144) | 119 (108-136) | 130 (126-138) | 122 (101-136) | 119 (115-125) | 0.642^c^ |
| Antithrombin (%) | 75-110 |  |  |  |  |  |  |
| All patients |  | 95 (84-105) | 92 (79-109) | 94 (75-106) | 111 (95-127)* | 107 (86-131)* | <0.001^a^ |
| SOFA$\leq10$ |  | 100 (87-111) | 102 (87-118) | 102 (96-134) | 115 (107-127) | 111 (101-135) | 0.006^b^ |
| SOFA >10 |  | 88 (78-102) | 81 (75-92)^#^ | 74 (64-91)^#^ | 97 (86-122) | 87 (77-108) | 0.021^c^ |
| Protein C (u/mL) | 60-130 |  |  |  |  |  |  |
| All patients |  | 78 (64-85) | 81 (66-92) | 89 (70-114)* | 139 (117-152)* | 131 (116-158)* | <0.001^a^ |
| SOFA$\leq10$ |  | 83 (72-90) | 90 (81-103) | 107 (85-132) | 147 (136-160) | 149 (131-168) | 0.001^b^ |
| SOFA >10 |  | 68 (52-80)^#^ | 68 (51-78)^#^ | 61 (55-89)^#^ | 116 (102-139)^#^ | 128 (114-140) | 0.015^c^ |
| Free Protein S (u/mL) | 55-140 |  |  |  |  |  |  |
| All patients |  | 29 (20-39) | 28 (20-48) | 47 (32-74)* | 76 (68-87)* | 87 (74-96)* | <0.001^a^ |
| SOFA$\leq10$ |  | 30 (23-47) | 35 (20-56) | 50 (38-86) | 80 (72-105) | 88 (72-93) | 0.086^b^ |
| SOFA >10 |  | 29 (20-38) | 28 (18-35) | 41 (18-65) | 71 (55-77) | 87 (76-105) | 0.082^c^ |

Values represent median (IQR). SOFA: sequential organ failure assessment score. P values were calculated with the use of generalized estimating equations (GEE): (a): time effect, (b): group effect and (c): time-group interaction. Pairwise comparisons significant at the 0.05 level: (*): time effect - pooled patients: each time point vs. Day 0. (#): between group comparisons (group SOFA>10 vs. group SOFA ≤10) at each time point.
