# Supplementary material for: Coagulation profile of COVID-19 patients admitted to the ICU: An exploratory study
Source: PLoS One. 2020 Dec 15;15(12):e0243604. doi: 10.1371/journal.pone.0243604 (PMC7737963; doi:10.1371/journal.pone.0243604)
Supplement: Attachment — Submitted filename: Response to Reviewers.docx [file pone.0243604.s002.docx]

**Dear Editor and Reviewers**

**Thank you very much for the interesting and important inputs to our study**

**PONE-D-20-25608 - COAGULATION PROFILE OF COVID-19 PATIENTS ADMITTED TO THE ICU: AN EXPLORATORY STUDY

Responses to the Editor**

**The study does not have a group of ICU patients to look for specific factors altered in COVID-19.**

We didn’t do a control group at the time of the study, as the study planning and even data collection occurred at the beginning of the pandemic in Brazil and the Hospital Israelita Albert Einstein, where the study was done, was reorganized to treat, almost exclusively, patients infected with COVID-19 as it happened around the world. We addressed such limitation in our text *“Secondly, during the study period, all the ICU beds available in our department were destinated to COVID-19 patients. Thus, inclusion of a control group without severe SARS-CoV-2 infection was not possible. Nevertheless, the coagulation profile of patients admitted to the ICU in our center has been recently addressed [15]*” The reference cited above (#15) evaluated 531 critical ill patients and showed normal ROTEM profilte in the majority of ICU, non COVID-19, patients.

Also, to make it clearer, we included this sentence following the previous paragraph “*Also, a recent study have already demonstrated a higher incidence of thrombotic complications were diagnosed in COVID-19 ARDS patients than in patients with non-COVID-19 ARDS*”. (included refence number 5 already cited in our study)

So, it was not possible to include a control group during the study period, only on July the pandemic started to be more controlled in our city and we returned to have non-COVID-19 patients in our ICU. By the reasons discussed, it would have taken a long time to have had a control group and then delay the manuscript submission.

**The authors should provide convincing published evidence in order to understand in which way the altered parameters are specific of COVID-19**

We agree that there are indirect effects of any infection, such as through severe illness with subsequently inflammatory response that may predispose patients to thrombotic events and this is not specific for COVID-19 patients as well as hypoxia can contribute to thrombotic events and again it is not specific for hypoxemic COVID-19 patients. However, COVID-19 patients demonstrated a higher incidence of thrombotic events that highlighted the importance of better understand its specific changes in the coagulation system and it is the main objective of our study. The following epidemiological studies highlighted the greater incidence of thrombosis events in patients hospitalized with COVID-19 specially in ICU patients

1. Klok FA, Kruip M, van der Meer NJM, Arbous MS, Gommers D, Kant KM, et al. Incidence of thrombotic complications in critically ill ICU patients with COVID-19. Thrombosis research. 2020;191:145-7.
2. Helms J, Tacquard C, Severac F, Leonard-Lorant I, Ohana M, Delabranche X, et al. High risk of thrombosis in patients with severe SARS-CoV-2 infection: a multicenter prospective cohort study. Intensive care medicine. 2020;46(6):1089-98.
3. Lodigiani C, Iapichino G, Carenzo L, Cecconi M, Ferrazzi P, Sebastian T, et al. Venous and arterial thromboembolic complications in COVID-19 patients admitted to an academic hospital in Milan, Italy. Thrombosis research. 2020;191:9-14.
4. Bikdeli B, Madhavan MV, Jimenez D, Chuich T, Dreyfus I, Driggin E, et al. COVID-19 and Thrombotic or Thromboembolic Disease: Implications for Prevention, Antithrombotic Therapy, and Follow-Up: JACC State-of-the-Art Review. Journal of the American College of Cardiology. 2020;75(23):2950-73.
5. Klok FA, et al. .[Confirmation of the high cumulative incidence of thrombotic complications in critically ill ICU patients with COVID-19: An updated analysis.](https://pubmed.ncbi.nlm.nih.gov/32381264/) Thromb Res. 2020. PMID: 32381264

We included the further sentece, on the Discussion section, to address the correlation of COVID-19 and thrombosis profile of patients with COVID-19: “*There are some pathophysiological changes secondary to COVID-19 infection that may contribute to a greater chance of patients infected with COVID-19 to develop thrombotic complications as an increased angiotensin II expression secondary to angiotensin-converting enzyme 2 receptor binding and consequently augmented plasminogen activator inhibitor C-1 expression with a reduced fibrinolysis in the anticoagulation system*. (**reference included:** Dolhnikoff M, Duarte-Neto  AN, de Almeida Monteiro  RA,  et al.  *Pathological evidence of pulmonary thrombotic phenomena in severe COVID-19*.  Thromb Haemost 2020 Jun;18(6):1517-1519.)

*Further, angiotensin II–mediated pulmonary vasoconstriction can predispose to stasis and hypercoagulability, as can COVID-19 induction of antiphospholipid antibodies and complement during cytokine storms, causing vasculitis and microthromboses*. (**reference included:** Medcalf RL, Keragala CB, Myles PS. *Fibrinolysis and COVID-19: a plasmin paradox*. J Thromb Haemost. 2020.)

**Heparin could affect ROTEM measurements**

We agree with this statement and it is one of the reasons that we changed in the Discussion section where it was included on the limitation paragraph: *Third, all patients were already receiving anticoagulants as DVT prophylaxis or systemic anticoagulation and these could change the ROTEM results.*

**Concerning the comments regarding protein S:**

We changed on Material and Methods section at the Laboratory analysis subsection “Fibrinolysis and endogenous anticoagulation system” that we measured the free protein S and in the Table and when it was cited in the text *we changed in the manuscript and always wrote “free protein S” to be more exact*.

We understand your comment about the importance of total protein S but protein S is a vitamin K-dependent glycoprotein, which acts as a cofactor for Activated Protein C, increasing its anticoagulant and profibrinolytic effects. Protein S is present in plasma in two forms: free protein S (40%) and protein S bound to the transport protein of the complement C4b fraction (60%). The two forms are in dynamic equilibrium and only free Protein S has biological activity. These are the reasons that we decided to include free protein S since its biological activity.

References to our decision:

1. Suzuki K, Nishioka J. Plasma Protein S Activity measured using Protac, a Snake Venom Derived Activator of Protein C, Thromb. Res. 1988; 49: 214-251.
2. Faioni EM, Valsecchi C, Palla A, Taioli E, Razzari C, Mannucci PM. Free protein S deficiency is a Risk Factor for Venous Thrombosis. Thromb Haemost 1997; 78: 1343-1346

**Response to Journal Requirements:**

When submitting your revision, we need you to address these additional requirements. Please ensure that your manuscript meets PLOS ONE's style requirements, including those for file naming.

 	We did the changed above according to PLOS ONE's style requirements

In your Methods section, please provide additional information about the participant recruitment method and the demographic details of your participants. Please ensure you have provided sufficient details to replicate the analyses such as:
a) the recruitment date range (month and year),
b) a statement as to whether your sample can be considered representative of a larger population, and
c) a description of how participants were recruited.

 We included the paragraph on the Methods Section: “Participants were recruited between March 29, 2020 through May 13, 2020 and they could represent the majority of severe patients infected by COVID-19, once there were few exclusion criteria and the participants were recruited with waiver of informed consent once it was an observational study without any intervention and consecutive patients admitted in the Intensive Care Unit were recruited following the inclusion and exclusion criteria until we completed 30 patients included in the study.”

**Response to the comments of the reviewers**

Have the authors made all data underlying the findings in their manuscript fully available?
The [PLOS Data policy](http://www.plosone.org/static/policies.action#sharing) requires authors to make all data underlying the findings described in their manuscript fully available without restriction, with rare exception (please refer to the Data Availability Statement in the manuscript PDF file). The data should be provided as part of the manuscript or its supporting information, or deposited to a public repository. For example, in addition to summary statistics, the data points behind means, medians and variance measures should be available. If there are restrictions on publicly sharing data—e.g. participant privacy or use of data from a third party—those must be specified.

 Reviewer #1: No.

We provided now all our dataset, in the *datadryad.org* following the Plos One policy after your comment.

Please, find bellow the message from datadryad: “Your dataset has been assigned a unique identifier, called a DOI (doi:10.5061/dryad.pg4f4qrn3). If your dataset is associated with a manuscript submission, you may provide this DOI to the journal, although it will not be live until the dataset is published. For private access prior to publication, you may share your dataset using this temporary link: <https://datadryad.org/stash/share/iz0FlU7yXkYg4_Yh2PM_2y0cPIPTyiD6_lry0NMfJKM>.”

**Review Comments to the Author
Comments**

**No non-COVID19 ICU control group**

We agree that it should be interest to have a control group of ICU non-COVID-19 patients, but we didn’t do a control group at the time of the study, as the study planning and even data collection occurred at the beginning of the pandemic in Brazil and the Hospital Israelita Albert Einstein, where the study was done, was reorganized to treat, almost exclusively, patients infected with COVID-19 as it happened around the world. We addressed such limitation in our text *“Secondly, during the study period, all the ICU beds available in our department were destinated to COVID-19 patients. Thus, inclusion of a control group without severe SARS-CoV-2 infection was not possible. Nevertheless, the coagulation profile of patients admitted to the ICU in our center has been recently addressed [15]*”

The reference cited above (#15) evaluated 531 critical ill patients and showed normal ROTEM profilte in the majority of ICU, non COVID-19, patients.

Also, to make it clearer, we included this sentence following the previous paragraph “*Also, a recent study have already demonstrated a higher incidence of thrombotic complications were diagnosed in COVID-19 ARDS patients than in patients with non-COVID-19 ARDS*”. (included refence number 5 already cited in our study)

So, it was not possible to include a control group during the study period, only on July the pandemic started to be more controlled in our city and we returned to have non-COVID-19 patients in our ICU. By the reasons discussed, it would have taken a long time to have had a control group and then delay the

**I number of patients have been treated with UFH ie heparin. This may affect the ROTEM test.**

We agree with this statement and it is one of the reasons that we changed in the Discussion section where it was included on the limitation paragraph: *Third, all patients were already receiving anticoagulants as DVT prophylaxis or systemic anticoagulation and these could change the ROTEM results.*

Before the pandemic started, the ICU of Hospital Israelita Albert Einstein have already a DVT prophylaxis algorithm and a culture in the institution to introduce drug prophylaxis for DVT in critically ill patients, unless contraindicated, and even more so in patients with COVID-19, due to reports of initial studies already demonstrating a significant incidence of thrombotic events in such patients, then it would take too much time to include patients with COVID-19, whose prophylaxis for DVT was contraindicated, and then the study would take much time to be finished.

**It would have been nice to have a figures where the individuals patients values are plotted over time for the most important analysis at least.**

We included a figure

**Figure 2.** Fibrinolysis and endogenous inhibitors of coagulation.

Values represent median (IQR). Red lines represent group SOFA >10 (n=14), blue lines represent group SOFA ≤10 (n=16) and black dotted lines represent all patients (n=30). P values were calculated with the use of generalized estimating equations (GEE): (a): time effect, (b): group effect and (c): time-group interaction. Pairwise comparisons significant at the 0.05 level: (*): time effect - pooled patients: each time point vs. Day 0. (#): between group comparisons (group SOFA>10 vs. group SOFA ≤10) at each time point.

And we put the Table 5 as a supplemental material
**In table 3 and 4 it is unclear what the p-values actually is testing.**

We included the sentence in the Statistical analysis Section to clarify what p-values actually tested: “To account for longitudinal (repeated measurements) and correlated response continuous variables, between-group differences and within-group differences over time were assessed using generalized estimating equations (GEE), with group (SOFA ≤ 10 and group SOFA > 10) and study time points (time) as predictors. P values for group effect, time effect, and time-group interaction were presented. *When a time effect was detected in pooled patients, each time point (Day 1, 3, 7 and 14) was compared against Day 0. When a group effect or a time-group interaction were detected, between group comparisons (group SOFA>10 vs. group SOFA ≤10) were performed at each time point.* The Bonferroni method was used to account for multiple comparisons”

Also, we did some modifications on Table 3 to make it clearer and we changed the legend to the following phrase: “Values represent median (IQR). INR: international normalized ratio, aPTT: activated partial thromboplastin time. P values were calculated with the use of generalized estimating equations (GEE): (a): time effect, (b): group effect and (c): time-group interaction. *Pairwise comparisons significant at the 0.05 level: (*): time effect - pooled patients: each time point vs. Day 0. (#): between group comparisons (group SOFA>10 vs. group SOFA ≤10) at each time point*.”

We also changed the Table 4 as well as its legend to: “*Values represent median (IQR). SOFA: sequential organ failure assessment score. P values were calculated with the use of generalized estimating equations (GEE): (a): time effect, (b): group effect and (c): time-group interaction. Pairwise comparisons significant at the 0.05 level: (*): time effect - pooled patients: each time point vs. Day 0. (#): between group comparisons (group SOFA>10 vs. group SOFA ≤10) at each time point.”*

We also changed the Table 5, to a supplemental table as well as its legend to: “*Values represent median (IQR). SOFA: sequential organ failure assessment score. P values were calculated with the use of generalized estimating equations (GEE): (a): time effect, (b): group effect and (c): time-group interaction. Pairwise comparisons significant at the 0.05 level: (*): time effect - pooled patients: each time point vs. Day 0. (#): between group comparisons (group SOFA>10 vs. group SOFA ≤10) at each time point.”*

**There is a number of papers already published on this topic. What is the novelty with the present paper?**

**For instance: a) Hardy M, Douxfils J, Bareille M, Lessire S, Gouin-Thibault I, Fontana P, Lecompte T, Mullier F. Studies on hemostasis in COVID-19 deserve careful reporting of the laboratory methods, their significance and their limitations. J Thromb Haemost. 2020 Aug 13:10.1111/jth.15061**.

This is an interesting letter that mainly discussed limits of another study (Nougier C, Benoit R, Simon M, Desmurs-Clavel H, Marcotte G, Argaud L, et al. Hypofibrinolytic state and high thrombin generation may play a major role in sars-cov2 associated thrombosis. J Thromb Haemost. 2020. Online ahead of print. DOI: 10.1111/jth.15016.) showing the complexity in studying the coagulation profile of COVID-19 patients, but our study, different from this letter and also from the study cited above, analyzed evaluated different coagulation parameters on COVID-19 patient and for a longer period.

**b) Collett LW, Gluck S, Strickland RM, Reddi BJ. Evaluation of coagulation tatus using viscoelastic testing in intensive care patients with coronavirus disease 2019 (COVID-19): An observational point prevalence cohort study. Aust Crit Care. 2020 Jul 21:S1036-7314(20)30254-X.**

This is an interesting paper, but with a small sample (6 patients) and it evaluated mainly the ROTEM ^®^ profile and also only in a single measurement, our study, on the other hand, evaluated more coagulation examens and also during 14 days

**c) Creel-Bulos C, Auld SC, Caridi-Scheible M, Barker N, Friend S, Gaddh M, Kempton CL, Maier C, Nahab F, Sniecinski R. Fibrinolysis Shutdown and Thrombosis in A COVID-19 ICU. Shock. 2020 Aug 4**.

Similar to our study, this study also evaluated 21 patients, and it was demonstrated a fibrinolysis shutdown, but this study assessed mainly the ROTEM^®^ profile and also only in a single day and unlike our study that evaluated different coagulation parameters over time and also compared different clinical groups (SOFA < or > 10).

**d) Ibañez C, Perdomo J, Calvo A, Ferrando C, Reverter JC, Tassies D, Blasi A. High D dimers and low global fibrinolysis coexist in COVID19 patients: what is going on in there? J Thromb Thrombolysis. 2020 Jul 15:1–5.**

Similar to our study, this study also evaluated 19 patients, and it was also demonstrated a hypofibrinolytic pattern on the viscoelastic examen and elevated D-dimers value as we demonstrated, however this study above did not evaluated other coagulation parameters as we did and we demonstrated a time effect on ROTEM^®^ profile that was not demonstrated in the study above.

**e) Nougier C, Benoit R, Simon M, Desmurs-Clavel H, Marcotte G, Argaud L, David JS, Bonnet A, Negrier C, Dargaud Y. Hypofibrinolytic state and high thrombin generation may play a major role in SARS-COV2 associated thrombosis. J Thromb Haemost. 2020 Jul 15:10.1111/jth.15016. doi: 10.1111/jth.15016.**

It is a very interesting paper which the authors investigated several different coagulation parameters, however the patients included were less sicker with a lower mean SOFA score, lower rate of mechanical ventilation and also it was only done discussed one single day measurement of the coagulation profile, so it was not studied the coagulation behavior along two weeks as we done and our study the patients included were general more sicker.

**f)Pavoni V, Gianesello L, Pazzi M, Stera C, Meconi T, Frigieri FC. Evaluation of coagulation function by rotation thromboelastometry in critically ill patients with severe COVID-19 pneumonia. J Thromb Thrombolysis. 2020**

This study is very interesting, since as our study, the authors investigated the coagulation profile over 10 days (we investigated during two weeks) but the study above include less severe patients, where only 10% of the patients need mechanical ventilation, the mean SOFA was less than the less critical group form our study (group SOFA < 10), and there are not two groups as our study to evaluate the impact and relationship of the coagulation system disorders and the organ dysfunction in ICU COVID-19 patients, one issue that our study has addressed.

Thank you for sharing these important and interesting references.

We included in our Discussion section the following sentence: *“We believe that despite several studies addressing the alterations in the coagulation system of COVID-19 patients, (****included all the references above****) our study continues to bring important news because it was one of the few that evaluated the behavior of the coagulation system, of critically critical patients infected with COVID-19, during two weeks of hospitalization. Also, our study was one of the few where we tried to assess the impact of the degree of change in the coagulation system and its impact on the evolution of organ dysfunctions presented by patients during the two weeks of study”.*

**Reviewer #2: This is an intriguing study focused on an important topic linked to COVID-19.
The relevance of coagulation abnormalities in COVID-19 patients is clearly underscored by the relationship between degree of severity of disease and indicators of abnormal clotting.**

**It would be interesting to more clearly postulate in the discussion the mechanism by which the viral infection may lead to the reported findings.**

We included this sentence on the Discussion section to address this relationship: *“Several mechanics can explain the relationship between viral infection and our findings, as endothelial cell disruption, tissue factor expression, and activation of the coagulation cascade by cytokines released during viral infections are other possible mechanisms of thrombosis. This pro-inflammatory state can promote microthrombosis in the vascular lung system and consequently promoting more hypoxia with local impact creating a deleterious positive thromboinflammatory feedback loop* (**reference included**: Dolhnikoff M, Duarte-Neto  AN, de Almeida Monteiro  RA,  et al.  *Pathological evidence of pulmonary thrombotic phenomena in severe COVID-19.*  Thromb Haemost 2020 Jun;18(6):1517-1519. AND Wool GD, Miller JL. *The Impact of COVID-19 Disease on Platelets and Coagulation* Pathobiology. 2020 Oct 13:1-13. doi: 10.1159/000512007. AND Escher R, Breakey N, Lämmle B. *Severe COVID-19 infection associated with endothelial activation*. Thromb Res. 2020 Jun;190:62.)

**Furthermore, the role of Vitamin K abnormalities in patients with defective ROTEM should be discussed.**

We agree with the sentence above and is one of the reason that our exclusion criteria were, as cited in the manuscript: *“Exclusion criteria included pregnancy, previous known coagulopathy, currently use of systemic anticoagulants or anti-platelet therapy or* ***vitamin K antagonists****,* moribund patients and patients who presented cardiac arrests.

**Future investigations should attempt to validate the current observation in independent patients’ cohorts.**

We completely agree that it should be very interesting and important to validate the current observation in a ICU non COVID-19 population, however as explained in sentences above it would have taken a long time to have this population and we did not know when the peak of the pandemic in Brazil would be better allowing to return to a more stable situation where critical ill patients without COVID-19 infection would be hospitalized and we would be able to evaluate this population regarding deeply the coagulation system as we did in our study with severe ill COVID-19 patients. We intend to do this in a future research

**Finally, conducting a long-term follow up on patients with ROTEM severe abnormalities may provide further insights into the long-term vascular complications of the disease.**

We completely agree with this idea, and maybe we are going to evaluate for a longer period the coagulation system of critical ill ICU COVID-19 patients that stay more than 01 month in the ICU and/or hospital.

Thank you for your important inputs

Sincerely,

Ricardo Luiz Cordioli

26^TH^, October, 2020
