# Supplementary material for: Coagulation profile of COVID-19 patients admitted to the ICU: An exploratory study
Source: PLoS One. 2020 Dec 15;15(12):e0243604. doi: 10.1371/journal.pone.0243604 (PMC7737963; doi:10.1371/journal.pone.0243604)
Supplement: Attachment — Submitted filename: Response to Reviewers.docx [file pone.0243604.s003.docx]

**Dear Editor and Reviewers**

**Thank you very much for the interesting and important inputs to our study**

**PONE-D-20-25608 - COAGULATION PROFILE OF COVID-19 PATIENTS ADMITTED TO THE ICU: AN EXPLORATORY STUDY

Response to the comments of the reviewers**

1. The authors do not present in the abstract the increased d-dimer levels nor the transient decreased free protein S levels and plasminogen levels.

We changed the result paragraph on the Abstract Section:

*“Thirty patients were studied. Some conventional coagulation tests, as aPTT, PT and INR remained unchanged during the study period, while alterations on others coagulation laboratory tests were detected. Fibrinogen levels were increased in both groups. ROTEM maximum clot firmness increased in both groups from Day 0 to Day 14. Moreover, ROTEM – FIBTEM maximum clot firmness was high in both groups, with a slight decrease from day 0 to day 14 in group SOFA ≤ 10 and a slight increase during the same period in group SOFA > 10. Fibrinolysis was low and decreased over time in all groups, with the most pronounced decrease observed in INTEM maximum lysis in group SOFA > 10. Also, D-dimer plasma levels were higher than normal reference range in both groups and free protein S plasma levels were low in both groups at baseline and increased over time, Finally, patients in group SOFA > 10 had lower plasminogen levels and Protein C than patients with SOFA <10, which may represent less fibrinolysis activity during a state of hypercoagulability.”*

2. I do not agree with this sentence in the abstract ¨The hypercoagulability state of COVID-19 patients was only detected by ROTEM¨ or the sentences in discussion or conclusions:¨Finally, the hypercoagulability state of severe COVID-19 patients was detected by ROTEM, while conventional coagulation tests remained unchanged.¨ ¨The hypercoagulability state of patients infected with SARS-CoV-2 was detected by ROTEM but not with conventional coagulation tests.¨ Ordinary coagulation analysis showed increased fibrinogen, increased d-dimer, slightly increased antiplasmin, transient decreased free protein S. and slightly transient decreased plasminogen. Thus, not only ROTEM could identify a hypercoagulable state in COVID infection.

We changed the conclusion paragraph on the Abstract Section:

*“The hypercoagulability state of COVID-19 patients was not only detected by ROTEM but it much more complex, where changes were observed on the fibrinolytic and endogenous anticoagulation system.”*

We changed the paragraph on the Discussion Section:

*“Finally, the hypercoagulability state of severe COVID-19 patients was detected by ROTEM and modifications on some coagulation tests related to the fibrinolytic and endogenous anticoagulation system, while the more common conventional coagulation tests, as aPTT (sec) INR and platelets, remained unchanged.”*

We changed the paragraph on the Conclusion Section:

*“The hypercoagulability state of patients infected with SARS-CoV-2 was detected by ROTEM and other coagulation tests but not with usual coagulation tests. Our findings highlight the role of rotational thromboelastometry when monitoring the coagulation system in ICU patients with COVID-19 and also demonstrated that the mechanisms to explain the hypercoagulability state of patients infected with SARS-CoV-2 is very complex and need more studies”.*

3. A limitation is that the authors have not measured markers of hypercoagulability such as F1+2, TAT and endogenous thrombin potential. Nor have the authors measured any marker for endothelial dysfunction. I think this should be stated among limitations in the discussion. This has not only relevance for the authors conclusion that the hypercoagulability of COVID19 may be detected only by ROTEM and not conventional coagulation tests but also regarding the cause of hypercoagulability.

We agree with your important comment and we included in the limitation paragraph:

*“Fourth, we did not evaluate all factors involved in fibrinolysis, such as PAI-1 and plasmin, which preclude us to fully understand the role of fibrinolytic system on COVID-19 induced coagulopathy and also other laboratory tests as prothrombin fragment 1+2, thrombin-anti-thrombin complexes and endogenous thrombin potential assays were not done to better understand the hypercoagulability state of such patients. Finally, we not measured any marker for endothelial dysfunction which probably contribute to the modifications on the coagulation system of the severe patients infected with SARS-CoV-2. Nevertheless, our study was the first to demonstrate some aspects of coagulation disorders that can occur in critical patients infected by COVID-19, especially the deficiency of naturally anti-coagulant factors.”*

Thank you for your important inputs

Sincerely,

Ricardo Luiz Cordioli

19^TH^, November, 2020
